# Attempted Synthesis of *Vinca* Alkaloids Condensed with Three-Membered Rings

**DOI:** 10.3390/molecules23102574

**Published:** 2018-10-09

**Authors:** András Keglevich, Szabolcs Mayer, Réka Pápai, Áron Szigetvári, Zsuzsanna Sánta, Miklós Dékány, Csaba Szántay, Péter Keglevich, László Hazai

**Affiliations:** 1 Department of Organic Chemistry and Technology, University of Technology and Economics, Budapest, Hungary, H-1111 Budapest, Gellért tér 4., Hungary; keglevich.andras@mail.bme.hu (A.K.); mayer.szabolcs.g@gmail.com (S.M.); hazai@mail.bme.hu (L.H.); 2 ComInnex, Inc., Graphisoft Park (Building D), H-1031 Budapest, Záhony u. 7., Hungary; reka134@gmail.com; 3 Spectroscopic Research Department, Gedeon Richter Plc., H-1475 Budapest 10, P. O. Box 27, Hungary; szigetvaria@richter.hu (Á.S.); Zs.Santa@richter.hu (Z.S.); m.dekany@richter.hu (M.D.); cs.szantay@richter.hu (C.S.J.)

**Keywords:** halogencyclopropane, dichlorocarbene, epoxidation, vindoline, catharanthine, dimer alkaloids, vindoline trimer

## Abstract

Our successful work for the synthesis of cyclopropanated vinblastine and its derivatives by the Simmons–Smith reaction was followed to build up further three-membered rings into the 14,15-position of the vindoline part of the dimer alkaloid. Halogenated 14,15-cyclopropanovindoline was prepared by reactions with iodoform and bromoform, respectively, in the presence of diethylzinc. Reactions of dichlorocarbene with vindoline resulted in the 10-formyl derivative. Unexpectedly, in the case of the dimer alkaloids vinblastine and vincristine, the rearranged products containing an oxirane ring in the catharanthine part were isolated from the reactions. The attempted epoxidation of vindoline and catharanthine also led to anomalous rearranged products. In the epoxidation reaction of vindoline, an *o*-quinonoid derivative was obtained, in the course of the epoxidation of catharanthine, a hydroxyindolenine type product and a spiro derivative formed by ring contraction reaction, were isolated. The coupling reaction of vindoline and the spiro derivative obtained in the epoxidation of catharanthine did not result in a bisindole alkaloid. Instead, two surprising vindoline trimers were discovered and characterized by NMR spectroscopy and mass spectrometry.

## 1. Introduction

**Figure 1:**
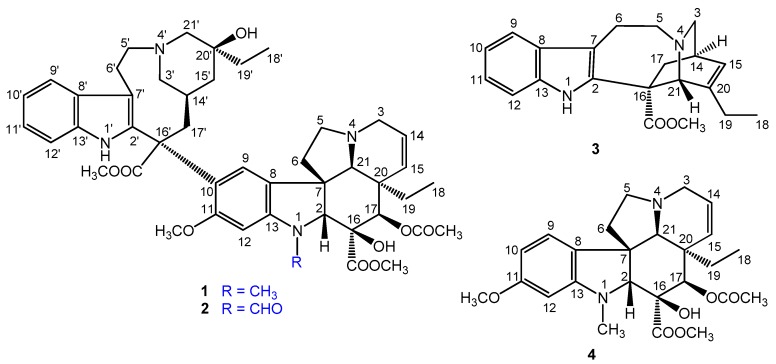
The alkaloids vinblastine (**1**), vincristine (**2**), catharanthine (**3**) and vindoline (**4**).

The “dimer alkaloids” vinblastine (vincaleukoblastine, VLB, **1**) and vincristine (VCR, **2**) (Figure 1), isolated from the Madagascar periwinkle *Catharanthus roseus*, belong to the family of the so-called “bisindole” alkaloids [1,2,3,4,5]. Their structure comprises two indole-related “monomers”: derivatives of catharanthine (**3**) and vindoline (**4**). VLB (**1**), VCR (**2**) and some of their analogs are anti-microtubule drugs that have been playing an important role in cancer chemotherapy for decades [6,7,8,9,10], and there are still many ongoing medicinal chemistry projects targeted at synthesizing new derivatives to improve the therapeutic effect of this family of drugs by increasing their selectivity or reducing their toxicity [11,12].

**Figure 2:**
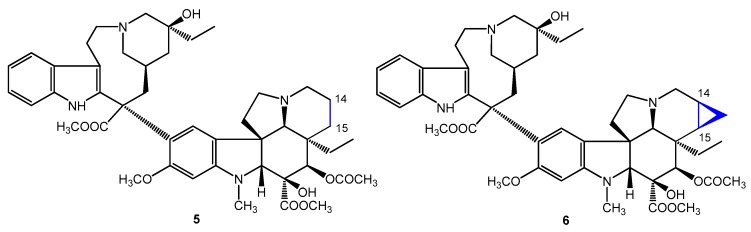
The alkaloids 14,15-dihydrovinblastine (**5**) and 14,15-cyclopropanovinblastine (**6**).

It is interesting to note that relatively minor changes in the bisindole structure can result in major changes in the biological activity. For example, one of the simplest such modification is the catalytic hydrogenation of the olefinic bond in the vindoline part of VLB (**1**), leading to 14,15-dihydrovinblastine (**5**), whose antitumor activity is significantly lower than that of VLB (**1**) (Figure 2) [13]. On the other hand, if instead of only being saturated, there is also a methylene bridge connecting C-14 and C-15, the resulting compound’s bioactivity changes dramatically. In our previous work [14,15,16] we synthesized different kinds of bisindole alkaloids condensed with a cyclopropane ring at position 14,15 of the vindoline part, namely cyclopropanovinblastine (**6**), cyclopropanovincristine, cyclopropanovinorelbin, and other derivatives. We reported their significant tumor cell inhibiting activity on different tumor types and tumor cell lines.

As a continuation of that work, our objective is the synthesis of further derivatives of potentially cytotoxic dimer alkaloids condensed with, e.g., a halogen substituted cyclopropane or oxirane ring. In our research group, these molecules have been a missing link in the analysis of the structure-activity relationship of the *Vinca* alkaloids. Synthetic studies were not only performed on the dimer alkaloids, but also on their building blocks, i.e*.*, catharanthine (**3**) and vindoline (**4**).

## 2. Results and Discussion

### 2.1. Generation of Halogencyclopropane Ring with the Simmons–Smith Reaction

**Scheme 1:**
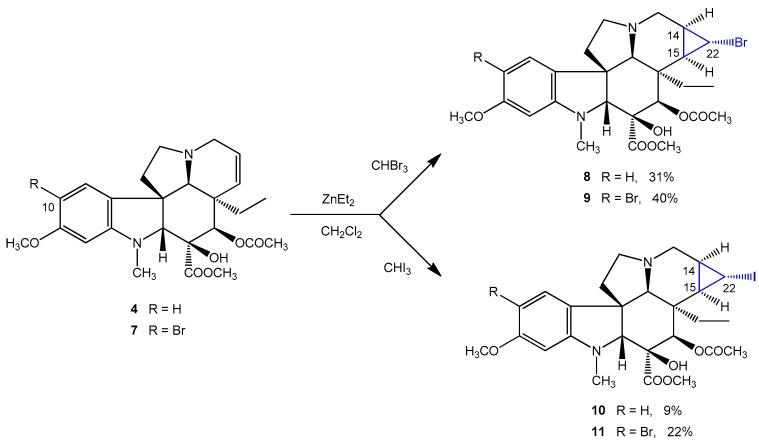
The formation of the bromo- and iodocyclopropane ring.

Our first purpose was to build up the halogencyclopropane ring in place of the C(14)=C(15) carbon-carbon double bond of the vindoline ring (**4**). Based on the work of Beaulieu et al*.* [17], vindoline (**4**) was reacted with bromoform and iodoform in the presence of diethylzinc in dichloromethane (Scheme 1). The Simmons–Smith reaction resulted stereospecifically in **8** 14,15-bromocyclopropanovindoline (31% yield) and **10** 14,15-iodocyclopropanovindoline (9% yield). By achieving the reaction with 10-bromovindoline (**7**) [18], the **9** bromocyclopropane and **11** iodocyclopropane derivatives were obtained in yields of 40% and 22%. The configurations of the 14, 15, and 22 carbon atoms were determined by NMR spectroscopy (14:(*S*), 15:(*R*), and 22:(*S*)).

### 2.2. Reactions with Dichlorocarbene

The following step of our current work was the investigation of the reactions of *Vinca* alkaloids with dichlorocarbene. Dichlorocyclopropano derivatives of monomeric and dimeric alkaloids were aimed at as an extension of our previous work.

**Scheme 2:**
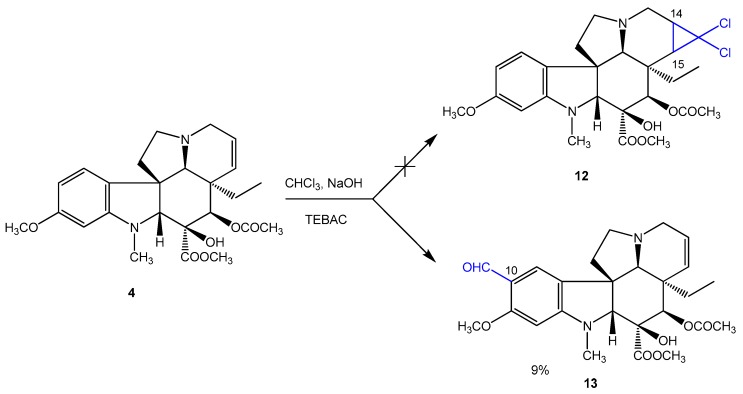
The classical method of the dichlorocarbene reaction was tried on vindoline (**4**) first.

First, the classical method of the reaction of dichlorocarbene with alkenes [19] was tried on vindoline (**4**) as a model compound (Scheme 2). A chloroform solution of vindoline (**4**) was treated with an aqueous solution of sodium hydroxide at room temperature in the presence of the phase transfer catalyst benzyltriethylammonium chloride (TEBAC). Unexpectedly, the dichlorocyclopropano derivative (**12**) could not be detected in the reaction. However, a formyl group was built in at position 10, resulting in 10-formylvindoline (**13**), a compound that is already known in the literature [20,21,22].

**Scheme 3:**
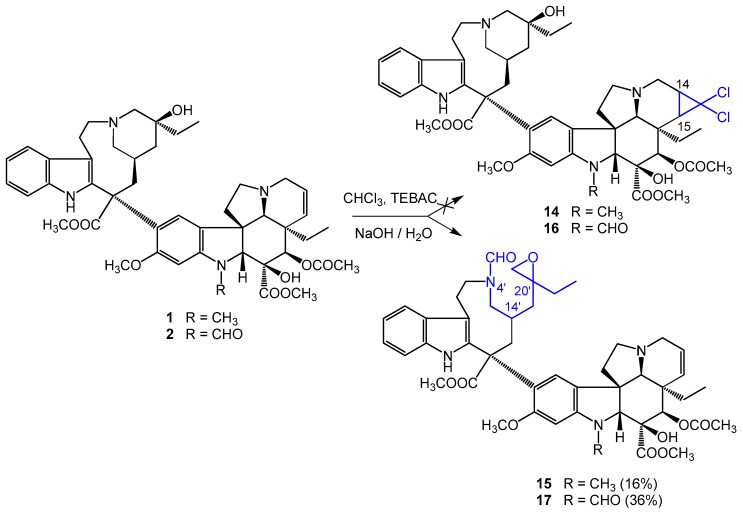
The dichlorocarbene reactions on the dimeric alkaloids vinblastine (**1**) and vincristine (**2**) led to ring-opened oxirane derivatives (**15** and **17**).

The above-mentioned dichlorocarbene reaction on the dimeric alkaloids vinblastine (**1**) and vincristine (**2**) was also investigated. In these reactions, instead of the expected dichlorocarbene adducts **14** and **16**, interesting ring-opened oxirane derivatives (**15** and **17**) were isolated in various yields (Scheme 3).

**Scheme 4:**
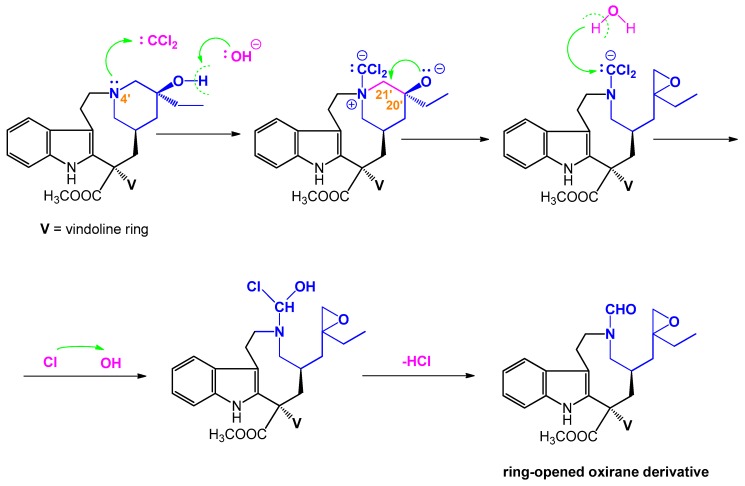
The proposed mechanism for oxirane formation.

A plausible interpretation of the oxirane formation is outlined in Scheme 4 (where **V** can represent non-modified and different kinds of modified “vindoline” units as well). First, electrophilic dichlorocarbene attacks the nucleophilic tertiary amine nitrogen at position 4’, leading to an ammonium compound as the intermediate. Then the hydroxyl group at C-20’ might become deprotonated under basic reaction conditions, the anionic moiety attacks C-21’, and N-4’ is neutralized during a heterolytic cleavage of a carbon-nitrogen bond. Finally, one of the chloro groups may undergo substitution with OH, and a formyl group forms by the elimination of hydrogen chloride.

### 2.3. Attempted Epoxidation of Vinca Alkaloid Building Blocks

Another objective of our research was the exploration of the reactivity of “monomeric” alkaloids (e.g., vindoline, catharanthine) towards epoxidizing agents. In the literature, only few oxirane derivatives of *Vinca* alkaloids are known. For instance, the 3-oxo derivative of catharanthine could be epoxidized with *m*-chloroperoxybenzoic acid [23]. Another example, mehranine, the 14,15-epoxi derivative of aspidospermidine, was isolated from *Tabernaemontana bovina* and its total synthesis was recently presented [24].

The vindoline and catharanthine derivatives bearing an oxirane ring were also tried to be obtained in similar reactions, and they it was attempted to use them in coupling reactions to afford epoxidized bisindole alkaloids.

**Scheme 5:**
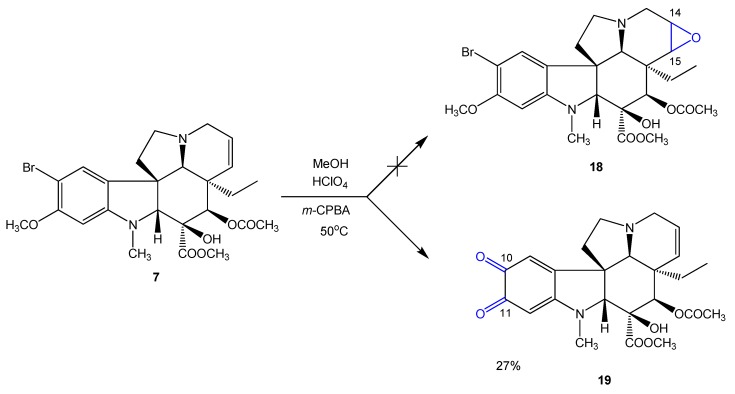
The attempted epoxidation of 10-bromovindoline (**7**) in the presence of perchloric acid.

First, the oxidation of 10-bromovindoline (**7**) with *m*-chloroperoxybenzoic acid (*m*-CPBA) was studied. The reaction could be performed either in the presence or absence of perchloric acid, which may influence the outcome of the oxidation [25]. When carrying out the reaction in methanol without HClO_4_, none of the oxidated products could be isolated. Surprisingly, when HClO_4_ was used in the reaction, the debromination and demethylation at the aromatic ring occurred. An *o*-quinoidal compound (**19**) was isolated in 27% yield, but the desired oxirane **18** could not be obtained (Scheme 5). Starting from compound **19**, the reaction has been described in a short article published last year by us [26].

**Scheme 6:**
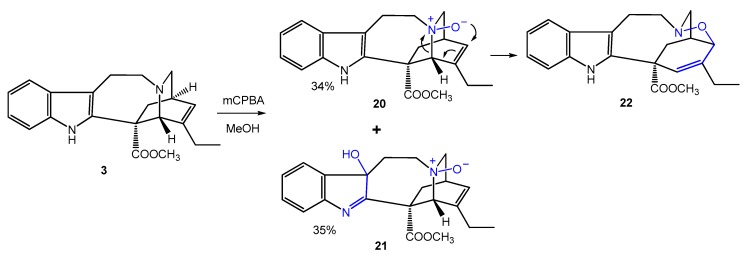
The oxidation with *m*-chloroperoxybenzoic acid was tried on catharanthine (**3**) without perchloric acid for the first time.

Hereafter the above-mentioned two methods of oxidation with *m*-CPBA were also tried on catharanthine (**3**). In the absence of perchloric acid, the oxidation led to a mixture of two products: catharanthine *N*^4^-oxide (**20**) in 34% yield (a similar preparation of **20** is known from the literature [18]), and a new compound (*N*^4^-oxide of 7-hydroxyindolenine catharanthine, **21**) in 35% yield (Scheme 6).

*N*-Oxide **20** is known to be unstable, it easily undergoes a [2,3]-sigmatropic rearrangement, leading to isoxazolidine **22** [27,28]. This kind of rearrangement during the NMR measurements (room temperature, CDCl_3_ as solvent) was also observed. Although Langlois et al. provided ^1^H and ^13^C NMR spectral data for **22** [19], they could not give an unequivocal assignment of the NMR peaks. In this paper, we report the unambiguous ^1^H and ^13^C-NMR assignments for **22**, which has become achievable because of the advancements in NMR instrumentation and measurement techniques since 1976.

**Scheme 7:**
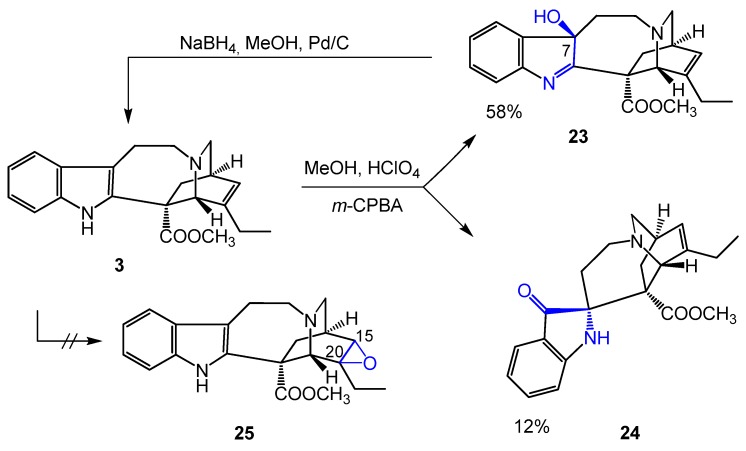
The oxidation with *m*-chloroperoxybenzoic acid was tried on catharanthine (**3**) in the presence of perchloric acid as well.

On the other hand, when perchloric acid is applied during the oxidation of catharanthine (**3**) with *m*-CPBA, two anomalous products were obtained. The major product (58% yield) was 7β-hydroxyindolenine catharanthine (**23**), the minor product (12% yield) was the spiro derivative **24** (Scheme 7).

The synthesis of 7β-hydroxyindolenine catharanthine (**23**) is already known [29]. The authors used singlet oxygen to oxidize catharanthine (**3**). They determined the configuration of compound **23** at C-7 by NMR spectroscopy using nuclear Overhauser effect (NOE) measurements. Our independent NMR assignments, including the determination of the relative stereochemistry of our product, were in full agreement with those reported in Reference [29]. Hydroxyindolenines are also natural products, e.g., the 15,20-dihydro derivative of **23** is known as coronaridine hydroxyindolenine and was isolated from *Ervatamia coronaria* var. *plena* [30].

To utilize **23** in an oxidative coupling reaction [22] with vindoline (**4**) with the purpose of obtaining new bisindole alkaloids, the double bond at N-1–C-2 has to be saturated, supposing that the coupling reaction requires a secondary amino group (i.e., R^1^R^2^NH) on the catharanthine site. Thus, a reduction of **23** was aimed at with sodium borohydride to obtain its 1,2-dihydro derivative, but the reaction resulted in the elimination of the hydroxyl group, returning it to catharanthine (**3**).

**Figure 3:**
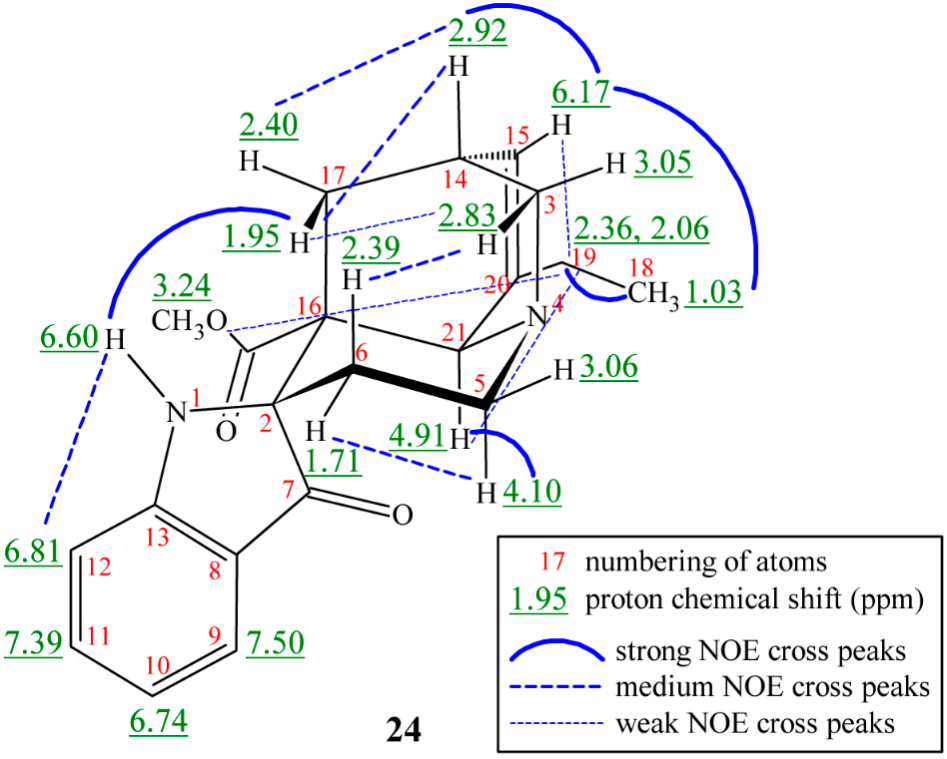
The three-dimensional structure of the spiro derivative of catharanthine (**24**).

Rosamine, a natural product isolated from *Catharanthus roseus*, was reported by Atta-ur-Rahman et al*.* with the same constitution as **24** [31], although they did not specify the configuration of any of the stereogenic centers. We determined the relative configuration of **24** based on NOE measurements (Figure 3), but we could not decide if **24** was rosamine. The reason for that was the unavailability of reliable spectral data for rosamine (in Reference [31], only the ^1^H NMR chemical shifts and coupling constants for the methyl groups and H-21 were given), so a reliable comparison of the spectral data of **24** with those of rosamine was not possible.

The stereogenic centers of **24** are C-2, N-4, C-14, C-16 and C-21 (Figure 3). Since **24** has a rigid cage structure (2-azabicyclo[2.2.2]octane) condensed with a six-membered ring, the relative configurations of N-4, C-14, C-16, and C-21 are fixed (the configuration at C-21 can be arbitrarily established as *R*, then N-4, C-14, and C-16 must be *R*,*R*,*R*). With this in mind, the configuration of C-2 was determined as follows. The NOESY correlation between NH-1 (6.60 ppm) and one of the protons at C-17 (1.95 ppm) implies that the configuration of C-2 is *R* (otherwise we would expect correlations between NH-1 and the protons at C-5), and the actual three-dimensional structure is supported by several other NOESY correlations (Figure 3).

### 2.4. Coupling Reaction of the Spiro Derivative of Catharanthine and Vindoline

**Scheme 8:**
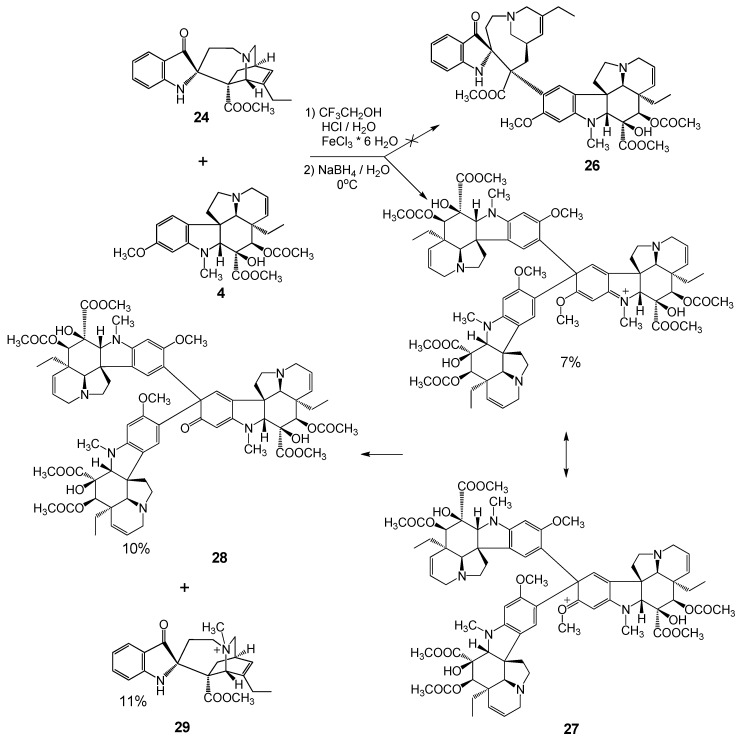
The coupling reaction of the spiro derivative of catharanthine (**24**) and vindoline (**4**) led to similar trimeric vindoline derivatives.

The coupling reaction of **24** with vindoline (**4**) was carried out according to a general procedure formerly used by our research group in the course of synthesizing VLB derivatives [14,15], which is an oxidation in the presence of trifluoroethanol and FeCl_3_ in acidic medium, followed by the reduction of the intermediate with sodium borohydride (Scheme 8).

Instead of the desired bisindole **26**, only unexpected products were found in the reaction mixture. We encountered that vindoline coupled to itself: a cationic trimeric vindoline (**27**) and a trimeric vindoline ketone (**28**) were isolated. The presence of a third product, *N*-methyl quaternary salt of a spiro derivative **29** suggests that the role of the spiro derivative **24** in the reaction might be the demethylation of the mesomer **27**. To support our assumption, the reaction was repeated without adding the spiro derivative of catharanthine (**24**). This way, only *O*-methylated trimeric vindoline (**27**) could be isolated and ketone **28** was not found in the reaction mixture, which substantiates our hypothesis.

## 3. Experimental Section

### 3.1. General

Vinblastine, vincristine, and catharanthine were derived from the corresponding sulfate salts; the bases were released directly before using them in the reactions. *m*-Chloroperoxybenzoic acid (*m*-CPBA) of 77% assay was purchased from Sigma-Aldrich (Budapest, Hungary) and was used as received. Melting points were measured on a VEB Analytik Dresden PHMK-77/1328 apparatus (Dresden, Germany) and are uncorrected. IR spectra were recorded on the Zeiss IR 75 and 80 instruments (Thornwood, NY, USA). NMR measurements were performed on a Varian VNMRS 800 MHz NMR spectrometer equipped with a ^1^H{^13^C/^15^N} Triple Resonance ^13^C Enhanced Salt Tolerant Cold Probe, a Varian VNMRS 500 MHz NMR spectrometer equipped with a ^1^H {^13^C/^15^N} 5 mm PFG Triple Resonance ^13^C Enhanced Cold Probe (Varian, Inc., Palo Alto, CA, USA), and a Bruker Avance III HDX 500 MHz NMR spectrometer equipped with a ^1^H {^13^C/^15^N} 5 mm TCI CryoProbe (Bruker Corporation, Billerica, MA, USA). ^1^H And ^13^C chemical shifts are given on the delta scale as parts per million (ppm) with tetramethylsilane (TMS) (^1^H, ^13^C) or dimethylsulfoxide-*d_6_* (^13^C) as the internal standard (0.00 ppm and 39.4 ppm, respectively). ^1^H-^1^H, direct ^1^H-^13^C, and long-range ^1^H-^13^C scalar spin-spin connectivites were established from 2D COSY, TOCSY, HSQC, and HMBC experiments. ^1^H-^1^H spatial proximities were determined using either two-dimensional NOESY/ROESY experiments or their selective 1D counterparts. All pulse sequences were applied by using the standard spectrometer software package. All experiments were performed at 298 K. NMR spectra were processed using VnmrJ 2.2 Revision C (Varian, Inc. Palo Alto, CA, USA), Bruker TopSpin 3.5 pl 6 (Bruker Corporation, Billerica, MA, USA) and ACD/Spectrus Processor version 2017.1.3 (Advanced Chemistry Development, Inc., Toronto, ON, Canada). HRMS and MS analyses were performed on an LTQ FT Ultra as well as an LTQ XL (Thermo Fisher Scientific, Bremen, Germany) system. The ionization method was ESI operated in the positive ion mode. For the CID (collision-induced dissociation) experiment, helium was used as the collision gas, and the normalized collision energy (expressed in percentage), which is a measure of the amplitude of the resonance excitation RF voltage applied to the endcaps of the linear ion trap, was used to induce fragmentation. The protonated molecular ion peaks were fragmented by CID at a normalized collision energy of 35–55%. The samples were dissolved in methanol. Data acquisition and analysis were accomplished with Xcalibur software version 2.0 (Thermo Fisher Scientific, Bremen, Germany). TLC was carried out using Kieselgel 60F_254_ (Merck, Budapest, Hungary) glass plates.

### 3.2. Bromocyclopropanation of Vindoline *(**4**)*

Vindoline (**4**) (228 mg, 0.50 mmol) was dissolved in dichloromethane (5 mL) and under Ar with 1.28 mL (1.28 mmol in 1 *M* hexane solution) of diethylzinc and then 88 μL (1.00 mmol) bromoform was injected to the solution. After stirring for 2 h at room temperature the reaction mixture was filtered and the filtrate was diluted with dichloromethane (50 mL) and washed with water (100 mL). The aqueous phase was extracted with dichloromethane (4 × 50 mL) and the combined organic phase was dried over magnesium sulfate, filtered, and the filtrate was evaporated to dryness. The crude product was separated by preparative TLC (dichloromethane-methanol 19:1) and 86 mg (31%) of product (**8**) was isolated. Mp 118–119 °C.

TLC (dichloromethane-methanol 20:1); *R_f_* = 0.71.

IR (KBr) 2963, 1744, 1617, 1502, 1230, 1039 cm^−1^.

^1^H NMR (499.9 MHz, CDCl_3_) *δ* (ppm) 0.68 (t, *J* = 7.3 Hz, 3H, H_3_-18), 0.97 (dd, *J* = 9.7, 3.8 Hz, 1H, H-15), 1.00 (dq, *J* = 14.4, 7.3 Hz, 1H, H_x_-19), 1.62 (dddd *J* = 9.7, 3.8, 3.5, 0.7 Hz, 1H, H-14), 1.91 (dq, *J* = 14.4, 7.3 Hz, 1H, H_y_-19), 2.21 (s, 3H, C(17)-OC(O)C*H_3_*), 2.22–2.40 (m, 5H, H_2_-6, H-21, H_x_-3, H_x_-5), 2.63 (s, 3H, N(1)-C*H_3_*), 3.23–3.33 (br m, 1H, H_y_-5), 3.44 (br d, *J* = 11.4 Hz, 1H, H_y_-3), 3.52 (t, *J* = 3.8 Hz, 1H, H-22), 3.57 (s, 1H, H-2), 3.78 (s, 3H, C(11)-OC*H_3_*), 3.81 (s, 3H, C(16)-COOC*H_3_*), 5.53 (s, 1H, H-17), 6.07 (d, *J* = 2.2 Hz, 1H, H-12), 6.30 (dd, *J* = 8.2, 2.2 Hz, 1H, H-10), 6.84 (d, *J* = 8.2 Hz, 1H, H-9), 7.96 (br s, 1H, C(16)-O*H*).

^13^C NMR (125.7 MHz, CDCl_3_) *δ* (ppm) 8.2 (C-18), 21.6 (C(17)-OC(O)*C*H_3_), 23.3 (C-14), 23.5 (C-22), 28.0 (C-15), 34.4 (C-19), 38.7 (N(1)-*C*H_3_), 41.8 (C-20), 44.6 (C-6), 51.9 (C-3), 52.2 (C-7), 52.7 (C(16)-COO*C*H_3_), 53.5 (C-5), 55.6 (C(11)-O*C*H_3_), 69.6 (C-21), 76.3 (C-17), 78.9 (C-16), 83.7 (C-2), 95.9 (C-12), 105.1 (C-10), 122.7 (C-9), 125.6 (C-8), 153.7 (C-13), 161.2 (C-11), 171.2 (C(17)-O*C*(O)CH_3_), 171.9 (C(16)-*C*OOCH_3_).

HRMS: M + H = 549.15965 (C_26_H_34_O_6_N_2_Br, Δ = 0.3 ppm). HR-ESI-MS-MS (CID = 35%, rel. int. %): 489(100); 457(1); 429(1); 314(1).

### 3.3. Bromocyclopropanation of 10-Bromovindoline *(**7**)*

The compound 10-Bromovindoline (**7**) (268 mg, 0.50 mmol) was dissolved in dichloromethane (5 mL) and under Ar with 1.28 mL (1.28 mmol in 1 *M* hexane solution) of diethylzinc and then 88 μL (1.00 mmol), the bromoform was injected to the solution. After stirring for 3 h at room temperature the reaction mixture was diluted with dichloromethane (40 mL) and washed with water (100 mL). The aqueous phase was extracted with dichloromethane (2 × 50 mL) and the combined organic phase was dried over magnesium sulfate, filtered and the filtrate was evaporated to dryness. The crude product was purified by preparative TLC (dichloromethane-methanol 30:1) and 126 mg (40%) of product (**9**) was isolated. Mp 144–146 °C.

TLC (dichloromethane-methanol 30:1); *R_f_* = 0.54.

IR (KBr) 2965, 1743, 1604, 1498, 1232, 1041 cm^−1^.

^1^H NMR (799.7 MHz, CDCl_3_) *δ* (ppm) 0.73 (t, *J* = 7.3 Hz, 3H, H_3_-18), 0.96–1.01 (m, 2H, H-15, H_x_-19), 1.66 (dt, *J* = 9.7, 3.5 Hz, 1H, H-14), 1.93 (dq, *J* = 14.4, 7.3 Hz, 1H, H_y_-19), 2.22–2.33 (m, 7H, C(17)-OC(O)C*H_3_*, H-21, H_2_-6, H_x_-3), 2.35–2.40 (m, 1H, H_x_-5), 2.66 (s, 3H, N(1)-C*H_3_*), 3.26–3.38 (br m, 1H, H_y_-5), 3.44–3.50 (br m, 1H, H_y_-3), 3.51 (br t, *J* = 3.5 Hz, 1H, H-22), 3.59 (s, 1H, H-2), 3.83 (s, 3H, C(16)-COOC*H_3_*), 3.90 (s, 3H, C(11)-OC*H_3_*), 5.53 (s, 1H, H-17), 6.10 (s, 1H, H-12), 7.07 (s, 1H, H-9), 7.98 (br, 1H, C(16)-O*H*).

^13^C NMR (201.1 MHz, CDCl_3_) *δ* (ppm) 8.1 (C-18), 21.7 (C(17)-OC(O)*C*H_3_), 23.2 br (C-14), 23.3 br (C-22), 27.9 (C-15), 34.5 (C-19), 38.7 (N(1)-*C*H_3_), 41.8 (C-20), 44.4 (C-6), 51.9 (C-3), 52.1 (C-7), 52.5 (C(16)-COO*C*H_3_), 53.4 (C-5), 56.3 (C(11)-O*C*H_3_), 69.5 (C-21), 76.1 (C-17), 78.7 (C-16), 83.6 (C-2), 94.5 (C-12), 100.2 (C-10), 126.1 (C-9), 126.5 br (C-8), 152.9 (C-13), 156.8 (C-11), 171.1 (C(17)-O*C*(O)CH_3_), 171.7 (C(16)-*C*OOCH_3_).

HRMS: M + H = 627.07035 (C_26_H_33_O_6_N_2_Br_2_, Δ = 0.6 ppm), HR-ESI-MS-MS (CID = 35%; rel. int. %): 567(100); 507(2).

### 3.4. Iodocyclopropanation of Vindoline *(**4**)*

Vindoline (**4**) (228 mg, 0.50 mmol) was dissolved in dichloromethane (20 mL) and under an Ar at 0 °C 1.28 mL (1.28 mmol in 1 *M* hexane solution) of diethylzinc, it was injected to the solution. Then 394 mg (1.00 mmol) of iodoform was added and the reaction mixture was stirred for 30 min at 0 °C and then for 6 h at room temperature. After allowing the solution to stand overnight, the addition of diethylzinc (1.28 mL) and iodoform (394 mg) was repeated at 0 °C. After stirring for 7 h at room temperature, the reaction mixture was filtered and the filtrate was diluted with dichloromethane (30 mL) and washed with water (100 mL). The aqueous phase was extracted with dichloromethane (5 × 60 mL) and the combined organic phase was dried over magnesium sulfate, filtered, and the filtrate was evaporated to dryness. The crude product was separated by preparative TLC (dichloromethane-methanol 19:1) and 28 mg (9%) of product (**10**) was isolated. Mp 116−117 °C.

TLC (dichloromethane-methanol 20:1); *R_f_* = 0.75.

IR (KBr) 2963, 1742, 1615, 1502, 1231, 1039 cm^−1^.

^1^H NMR (499,9 MHz, CDCl_3_) *δ* (ppm) 0.68 (t, *J* = 7.3 Hz, 3H, H_3_-18), 0.93 (dd, *J* = 9.2, 4.3 Hz, 1H, H-15), 0.99 (dq, *J* = 14.3, 7.3 Hz, 1H, H_x_-19), 1.52–1.57 (m, 1H, H-14), 1.89 (dq, *J* = 14.3, 7.3 Hz, 1H, H_y_-19), 2.21–2.31 (m, 7H, H_x_-3, H_2_-6, C(17)-OC(O)C*H_3_*, H-21), 2.32–2.41 (m, 1H, H_x_-5), 2.63 (s, 3H, N(1)-C*H_3_*), 3.13 (dd, *J* = 4.3, 3.7 Hz, 1H, H-22), 3.23–3.35 (m, 1H, H_y_-5), 3.42–3.50 (m, 1H, H_y_-3), 3.56 (s, 1H, H-2), 3.78 (s, 3H, C(11)-OC*H_3_*), 3.81 (s, 3H, C(16)-COOC*H_3_*), 5.53 (s, 1H, H-17), 6.07 (d, *J* = 2.3 Hz, 1H, H-12), 6.30 (dd, *J* = 8.2, 2.3 Hz, 1H, H-10), 6.83 (d, *J* = 8.2 Hz, 1H, H-9), 8.03 (br, 1H, C(16)-O*H*).

^13^C NMR (125.7 MHz, CDCl_3_) *δ* (ppm) -9.5 (C-22), 8.1 (C-18), 22.4 (C(17)-OC(O)*C*H_3_), 24.3 (C-14), 29.0 (C-15), 34.5 (C-19), 38.6 (N(1)-*C*H_3_), 42.1 (C-20), 44.6 (C-6), 52.2 (C-3), 52.3 (C-7), 52.4 (C(16)-COO*C*H_3_), 53.3 (C-5), 55.4 (C(11)-O*C*H_3_), 69.7 (C-21), 76.5 (C-17), 78.9 (C-16), 83.8 (C-2), 95.9 (C-12), 105.0 (C-10), 122.5 (C-9), 125.5 (C-8), 153.6 (C-13), 161.2 (C-11), 171.2 (C(17)-O*C*(O)CH_3_), 171.9 (C(16)-*C*OOCH_3_).

HRMS: M + H = 597.14664 (C_26_H_34_O_6_N_2_I, Δ = 1.7 ppm). HR-ESI-MS-MS (CID = 35%) (rel. int. %): 537(100); 505(1); 477(2); 441(1); 381(3); 362(2); 188(2).

### 3.5. Iodocyclopropanation of 10-Bromovindoline *(**7**)*

The compound 10-Bromovindoline (**7**) (268 mg, 0.50 mmol) was dissolved in dichloromethane (20 mL) and under Ar at 0 °C with 1.28 mL (1.28 mmol in 1 *M* hexane solution) of diethylzinc being injected into the solution. Then 394 mg (1.00 mmol) of iodoform was added and the reaction mixture was stirred for 30 min at 0 °C and then for 8 h at room temperature. After allowing the solution to stand overnight, the addition of diethylzinc (1.28 mL) and iodoform (394 mg) was repeated at 0 °C. After stirring for 6 h at room temperature, the reaction mixture was filtered and the filtrate was diluted with dichloromethane (30 mL) and washed with water (100 mL). The aqueous phase was extracted with dichloromethane (5 × 60 mL) and the combined organic phase was dried over magnesium sulfate, filtered and the filtrate was evaporated to dryness. The crude product was separated by preparative TLC (dichloromethane-methanol 19:1) and 74 mg (22%) of product (**11**) was isolated. Mp > 350 °C.

TLC (dichloromethane-methanol 20:1); *R_f_* = 0.86.

IR (KBr) 3444, 2927, 1740, 1228, 742 cm^−1^.

^1^H NMR (499,9 MHz, CDCl_3_) *δ* (ppm) 0.71 (t, 3H, *J* = 7.3 Hz, H_3_-18), 0.93 (dd, *J* = 9.2, 4.3 Hz, 1H, H-15), 0.93–1.01 (m, 1H, H_x_-19), 1.54–1.58 (m, 1H, H-14), 1.89 (dq, *J* = 14.6, 7.3 Hz, 1H, H_y_-19), 2.22–2.27 (m, 4H, H_x_-3, H_2_-6, H-21), 2.27 (s, 3H, C(17)-OC(O)C*H_3_*), 2.32–2.39 (m, 1H, H_x_-5), 2.64 (s, 3H, N(1)-C*H_3_*), 3.10 (t, *J* = 4.3 Hz, 1H, H-22), 3.24–3.30 (m, 1H, H_y_-5), 3.43–3.47 (m, 1H, H_y_-3), 3.57 (s, 1H, H-2), 3.81 (s, 3H, C(16)-COOC*H_3_*), 3.88 (s, 3H, C(11)-OC*H_3_*), 5.51 (s, 1H, H-17), 6.09 (s, 1H, H-12), 7.05 (s, 1H, H-9), 8.01 (br, 1H, C(16)-O*H*).

^13^C NMR (125.7 MHz, CDCl_3_) *δ* (ppm) -9.7 (C-22), 8.1 (C-18), 22.4 (C(17)-OC(O)*C*H_3_), 24.2 (C-14), 28.9 (C-15), 34.7 (C-19), 38.7 (N(1)-*C*H_3_), 42.1 (C-20), 44.5 (C-6), 52.17 (C-3), 52.22 (C-7), 52.5 (C(16)-COO*C*H_3_), 53.1 (C-5), 56.3 (C(11)-O*C*H_3_), 69.6 (C-21), 76.3 (C-17), 78.7 (C-16), 83.8 (C-2), 94.5 (C-12), 100.1 (C-10), 126.2 (C-9), 126.4 (C-8), 152.8 (C-13), 156.7 (C-11), 171.2 (C(17)-O*C*(O)CH_3_), 171.7 (C(16)-*C*OOCH_3_).

HRMS: M + H = 675.05411 (C_26_H_33_O_6_N_2_BrI, Δ = –2.9 ppm). ESI-MS-MS (CID = 35%) (rel. int. %): 615(100); 555(2); 459(1).

### 3.6. Attempted Dichlorocyclopropanation of Vindoline *(**4**)*

Vindoline (**4**) (256 mg, 0.56 mmol) and TEBAC (9 mg, 0.04 mmol) were dissolved in chloroform (1.4 mL), then a solution of sodium hydroxide (0.6 g, 15.00 mmol) in water (0.6 mL) was added dropwise to the reaction mixture, and stirred at room temperature for 2 h. The pH of the reaction mixture was adjusted to 7 using 1 M hydrochloric acid. Water was added and the mixture was extracted with chloroform. The combined organic phase was washed with water, dried over magnesium sulfate, and evaporated under reduced pressure. The residue was purified by preparative TLC (dichloromethane-methanol 20:1) and 24 mg (9%) of product (**13**) [20−22] was obtained.

### 3.7. Attempted Dichlorocyclopropanation of Vinblastine *(**1**)*

Vinblastine (**1**) (120 mg, 0.15 mmol) was dissolved in chloroform (0.4 mL), TEBAC (3 mg, 0.013 mmol) was added, and 200 mg (5.00 mmol) of NaOH in water (0.2 mL) was added dropwise. After stirring at room temperature for 2 h, the pH of the reaction mixture was neutralized using 1 M hydrochloric acid. Water (20 mL) was added and the mixture was extracted with chloroform (2 × 10 mL). The combined organic phase was dried over magnesium sulfate and evaporated under reduced pressure. The residue was purified by preparative TLC (dichloromethane-methanol 10:1) and 20 mg (16%) of product (**15**) was obtained. Mp 205−207 °C.

TLC (dichloromethane-methanol 10:1); *R_f_* = 0.60.

IR (KBr) 3470, 2964, 1740, 1669, 1614, 1501, 1461, 1371, 1227, 1040, 742 cm^−1^.

^1^H NMR (799.7 MHz, CDCl_3_) *δ* (ppm) 0.39 (t, *J* = 7.5 Hz, 3H, H_3_-18’), 0.82 (t, *J* = 7.4 Hz, 3H, H_3_-18), 0.91 (dd, *J* = 13.8, 12.3 Hz, 1H, H_x_-15’), 1.06 (dq, *J* = 14.7, 7.5 Hz, 1H, H_x_-19’), 1.36 (dq, *J* = 14.4, 7.4 Hz, 1H, H_x_-19), 1.46 (dd, *J* = 13.8, 3.4 Hz, 1H, H_y_-15’), 1.66 (dq, *J* = 14.7, 7.5 Hz, 1H, H_y_-19’), 1.83 (dq, *J* = 14.4, 7.4 Hz, 1H, H_y_-19), 1.99–2.05 (m, 1H, H-14’), 2.11 (s, 3H, C(17)-OC(O)C*H_3_*), 2.14 (ddd, *J* = 14.0, 8.7, 7.4 Hz, 1H, H_x_-6), 2.30 (br d, *J* = 16.5 Hz, 1H, H_x_-17’), 2.43 (d, *J* = 4.9 Hz, 1H, H_x_-21’), 2.44–2.47 (m, 1H, H_x_-5), 2.48 (dd, *J* = 13.2, 11.2 Hz, 1H, H_x_-3’), 2.55–2.56 (m, 1H, H_y_-21’), 2.62–2.66 (m, 2H, H-21, H_y_-6), 2.66 (s, 3H, N(1)-C*H_3_*), 2.79–2.83 (m, 1H, H_x_-3), 3.01–3.09 (br m, 1H, H_y_-17’), 3.15–3.20 (m, 1H, H_x_-6’), 3.31–3.35 (m, 1H, H_y_-5), 3.37–3.42 (m, 2H, H_x_-5’, H_y_-3), 3.50–3.61 (m, 2H, H_y_-6’, H_y_-5’) overlapped with 3.56 (br s, 3H, C(16’)-COOC*H_3_*), 3.73 (s, 1H, H-2), 3.80 (s, 3H, C(16)-COOC*H_3_*), 3.81 (s, 3H, C(11)-OC*H_3_*), 3.84–3.87 (m, 1H, H_y_-3’), 5.28–5.31 (m, 1H, H-15), 5.52 (s, 1H, H-17), 5.87 (ddd, *J* = 10.4, 4.8, 0.8 Hz, 1H, H-14), 6.11 (s, 1H, H-12), 6.65 (s, 1H, H-9), 7.10–7.14 (m, 2H, H-10’, H-12’), 7.16–7.19 (m, 1H, H-11’), 7.35 (br s, 1H, N(4’)-C*H*O), 7.51–7.53 (m, 1H, H-9’), 7.95 (br, 1H, NH-1’), 9.85 (br, 1H, C(16)-O*H*).

^13^C NMR (201.1 MHz, CDCl_3_) *δ* (ppm) 8.4 (C-18’), 8.5 (C-18), 21.2 (C(17)-OC(O)*C*H_3_), 24.9 (C-19’), 25.1 (C-6’), 28.8 (br, C-14’), 30.9 (C-19), 38.5 (N(1)-*C*H_3_), 39.1 (C-17’), 42.6 (C-15’), 42.7 (C-20), 43.6 (C-6), 49.5 (C-5’), 50.5 (C-3), 51.37 (C-5), 51.40 (C-3’), 52.2 (C(16)-COO*C*H_3_), 52.4 (C(16’)-COO*C*H_3_), 53.3 (C-7), 54.5 (C-21’), 55.7 (C(11)-O*C*H_3_), 56.4 (br, C-16’), 58.7 (C-20’), 66.4 (C-21), 76.6 (C-17), 79.7 (C-16), 84.0 (C-2), 94.0 (br, C-12), 110.8 (C-12’), 111.1 (br, C-7’), 117.6 (C-9’), 119.3 (C-10’), 120.0 (br, C-10), 122.5 (C-11’), 123.8 (br, C-8), 124.6 (C-14), 125.3 (C-9), 128.3 (C-8’), 129.9 (C-15), 133.0 (br, C-2’), 135.3 (br, C-13’), 153.3 (C-13), 157.9 (C-11), 163.4 (N(4’)-*C*HO), 171.0 (C(17)-O*C*(O)CH_3_), 171.8 (C(16)-*C*OOCH_3_), 174.4 (br, C(16’)-*C*OOCH_3_).

HRMS: M + Na = 839.41764 (C_45_H_60_O_10_N_4_Na, Δ = –3.0 ppm). ESI-MS-MS (CID = 35%, rel. int. %): 821(12), 779(100), 761(4), 747(4), 677(7), 570(45).

### 3.8. Attempt to Dichlorocyclopropanation of Vincristine *(**2**)*

Vincristine (**2**) (130 mg, 0.16 mmol) was dissolved in chloroform (1 mL) and TEBAC (7 mg, 0.031 mmol) was added. Then 230 mg (5.75 mmol) of NaOH in water (0.23 mL) was added dropwise. After stirring at room temperature for 2 h, the pH of the reaction mixture was neutralized with 1 M hydrochloric acid. Water (10 mL) was added and the mixture was extracted with dichloromethane (2 × 10 mL). The combined organic phase was dried over magnesium sulfate and evaporated under reduced pressure. The residue was purified by preparative TLC (dichloromethane-methanol 10:1) and 49 mg (36%) of product (**17**) was obtained. Mp 233–235 °C (decomp.).

TLC (dichloromethane-methanol 10:1); *R_f_* = 0.53.

IR (KBr) 3469, 1738, 1668, 1460, 1232, 744 cm^−1^.

NMR: two signal sets in a ratio of ca. 2:1 (conformational isomers due to hindered rotation of *N*-formyl group on the vindoline subunit of **2**).

Major signal set:

^1^H NMR (499.9 MHz, CDCl_3_) *δ* (ppm) 0.35–0.41 (m, 3H, H_3_-18’), 0.77 (br t, *J* = 7.3 Hz, 3H, H_3_-18), 0.97–1.06 (m, 2H, H_x_-19’, H_x_-15’), 1.26–1.43 (m, 3H, H_x_-19, H_y_-19’, H_y_-15’), 1.59–1.69 (m, 1H, H_y_-19), 1.88–1.98 (br m, 1H, H-14’), 2.01–2.08 (br m, 1H, H_x_-6) overlapped with 2.07 (br s, 3H, C(17)-OC(O)C*H_3_*), 2.33–2.44 (m, 2H, H_x_-17’, H_x_-21’), 2.45–2.54 (m, 2H, H_y_-21’, H_x_-3’), 2.54–2.70 (m, 2H, H_y_-6, H_x_-5), 2.84–2.93 (m, 2H, H_x_-3, H-21), 3.00–3.10 (br m, 1H, H_y_-17’), 3.17–3.22 (m, 1H, H_x_-6’), 3.35–3.43 (m, 3H, H_y_-3, H_y_-5, H_x_-5’), 3.46–3.53 (m, 1H, H_y_-6’), 3.53–3.60 (m, 1H, H_y_-5’), 3.62 (s, 3H, C(16’)-COOC*H_3_*), 3.73 (s, 3H, C(16)-COOC*H_3_*), 3.81 (br d, *J* = 13.7 Hz, 1H, H_y_-3’), 3.89 (br s, 3H, C(11)-OC*H_3_*), 4.76 (br s, 1H, H-2), 5.25 (br s, 1H, H-17), 5.42–5.46 (m, 1H, H-15), 5.91–5.95 (m, 1H, H-14), 6.80 (br s, 1H, H-12), 6.93 (br s, 1H, H-9), 7.09–7.15 (m, 1H, H-10’), 7.15–7.22 (m, 2H, H-12’, H-11’), 7.36 (br s, 1H, N(4’)-C*H*O), 7.51–7.55 (m, 1H, H-9’), 7.94 (br, 1H, NH-1’), 8.74 (s, 1H, N(1)-C*H*O), 9.23 (br, 1H, C(16)-O*H*).

^13^C NMR (125.7 MHz, CDCl_3_) *δ* (ppm) 8.1 (C-18, C-18’), 21.1 (C(17)-OC(O)*C*H_3_), 24.9 (C-19’), 25.0 (C-6’), 28.7 (br, C-14’), 30.7 (C-19), 38.6 (br, C-17’), 40.3 (C-6), 42.3 (C-20), 42.5 (C-15’), 49.5 (C-5’), 49.7 (C-5), 49.9 (C-3), 51.6 (C-3’), 52.6 (C(16’)-COO*C*H_3_), 52.9 (C(16)-COO*C*H_3_), 53.5 (C-7), 53.8 (C-21’), 56.1 (C(11)-O*C*H_3_), 56.7 (br, C-16’), 58.4 (C-20’), 65.0 (C-21), 72.6 (C-2), 77.0 (C-17), 79.8 (C-16), 94.8 (br, C-12), 111.1 (C-12’), 111.9 (C-7’), 117.7 (C-9’), 119.6 (C-10’), 122.8 (C-11’), 124.6 (C-8), 125.0 (C-14), 126.6 (br, C-10), 126.9 (C-9), 128.3 (C-8’), 129.6 (C-15), 132.1 (C-2’), 135.6 (C-13’), 141.7 (C-13), 157.7 (C-11), 160.1 (N(1)-*C*HO), 163.4 (N(4’)-*C*HO), 170.5 (C(17)-O*C*(O)CH_3_), 170.7 (C(16)-*C*OOCH_3_), 173.6 (br, C(16’)-*C*OOCH_3_).

Minor signal set:

^1^H NMR (499.9 MHz, CDCl_3_) *δ* (ppm) 0.35–0.41 (m, 3H, H_3_-18’), 0.64–0.70 (m, 3H, H_3_-18), 0.97–1.06 (m, 2H, H_x_-19’, H_x_-15’), 1.26–1.43 (m, 3H, H_x_-19, H_y_-19’, H_y_-15’), 1.59–1.69 (m, 1H, H_y_-19), 1.88–1.98 (br m, 1H, H-14’), 2.01–2.08 (br m, 1H, H_x_-6), 2.10 (br s, 3H, C(17)-OC(O)C*H_3_*), 2.33–2.44 (m, 2H, H_x_-17’, H_x_-21’), 2.45–2.54 (m, 2H, H_y_-21’, H_x_-3’), 2.54–2.70 (m, 2H, H_y_-6, H_x_-5), 2.84–2.90 (m, 1H, H_x_-3), 2.95 (br s, 1H, H-21), 3.00–3.10 (br m, 1H, H_y_-17’), 3.17–3.22 (m, 1H, H_x_-6’), 3.35–3.43 (m, 3H, H_y_-3, H_y_-5, H_x_-5’), 3.46–3.53 (m, 1H, H_y_-6’), 3.53–3.60 (m, 1H, H_y_-5’), 3.62 (s, 3H, C(16’)-COOC*H_3_*), 3.78 (s, 3H, C(16)-COOC*H_3_*), 3.81 (br d, *J* = 13.7 Hz, 1H, H_y_-3’), 3.90 (br s, 3H, C(11)-OC*H_3_*), 4.52 (br s, 1H, H-2), 5.29 (br s, 1H, H-17), 5.42–5.46 (m, 1H, H-15), 5.92–5.96 (m, 1H, H-14), 6.86 (br s, 1H, H-9), 7.09–7.15 (m, 1H, H-10’), 7.15–7.22 (m, 2H, H-12’, H-11’), 7.36 (br s, 1H, N(4’)-C*H*O), 7.51–7.55 (m, 1H, H-9’), 7.80 (br s, 1H, H-12), 7.96 (br, 1H, NH-1’), 8.21 (s, 1H, N(1)-C*H*O), 9.23 (br, 1H, C(16)-O*H*).

^13^C NMR (125.7 MHz, CDCl_3_) *δ* (ppm) 7.9 (C-18), 8.1 (C-18’), 21.1 (C(17)-OC(O)*C*H_3_), 25.0 (C-19’, C-6’), 28.7 (br, C-14’), 30.7 (C-19), 38.6 (br, C-17’), 40.0 (C-6), 42.3 (C-20), 42.4 (C-15’), 49.6 (C-5, C-5’), 50.0 (C-3), 51.7 (C-3’), 52.6 (C(16)-COO*C*H_3_), 52.9 (C(16’)-COO*C*H_3_), 53.1 (C-7), 53.8 (C-21’), 56.1 (C(11)-O*C*H_3_), 56.7 (br, C-16’), 58.4 (C-20’), 64.3 (C-21), 74.4 (C-2), 76.0 (C-17), 81.5 (C-16), 101.7 (br, C-12), 111.1 (C-12’), 111.7 (C-7’), 117.6 (C-9’), 119.5 (C-10’), 122.7 (C-11’), 124.3 (C-8), 125.1 (C-14), 125.7 (C-9), 126.6 (br, C-10), 128.2 (C-8’), 129.6 (C-15), 132.5 (C-2’), 135.6 (C-13’), 141.6 (C-13), 157.2 (C-11), 160.5 (N(1)-*C*HO), 163.4 (N(4’)-*C*HO), 170.3 (C(16)-*C*OOCH_3_), 170.4 (C(17)-O*C*(O)CH_3_), 173.7 (C(16’)-*C*OOCH_3_).

HRMS: M + Na = 853.39557 (C_45_H_58_O_11_N_4_Na, Δ = –4.5 ppm). ESI-MS-MS (CID = 35%, rel. int. %): 835(19), 811(7), 793(100), 775(4), 733(18), 705(3).

### 3.9. Epoxidation of 10-Bromovindoline *(**7**)*

To a solution of 10-bromovindoline (**7**) (300 mg, 0.56 mmol) in methanol (5 mL) and 72% perchloric acid (0.17 mL), *m*-CPBA (306 mg, 1.37 mmol) in methanol (2 mL) was added dropwise at 0 °C, and the reaction mixture was stirred at reflux for 5 h. Methanol was evaporated, 10% aqueous sodium carbonate solution (20 mL) was added to the residue, and the mixture was extracted with dichloromethane (3 × 20 mL). The combined organic phase was dried over magnesium sulfate and evaporated under reduced pressure. The residue was purified by preparative TLC (dichloromethane-methanol 15:1) and 70 mg (27%) of product (**19**) was obtained. Mp. 365 °C (decomp.).

TLC (dichloromethane-methanol 20:1); *R_f_* = 0.30.

IR (KBr) 3445, 1745, 1677, 1589, 1414, 1254 cm^−1^.

^1^H NMR (499.9 MHz, CDCl_3_) *δ* (ppm) 0.71 (t, *J* = 7.4 Hz, 3H, H_3_-18), 1.47 (dq, *J* = 14.8, 7.4 Hz, 1H, H_x_-19), 1.75 (dq, *J* = 14.8, 7.4 Hz, 1H, H_y_-19), 2.08 (s, 3H, C(17)-OC(O)C*H_3_*), 2.22 (ddd, *J* = 13.6, 11.2, 5.0 Hz, 1H, H_x_-6), 2.46 (ddd, *J* = 13.6, 9.0, 6.2 Hz, 1H, H_y_-6), 2.74 (ddd, *J* = 11.2, 9.8, 6.2 Hz, 1H, H_x_-5), 2.92–2.98 (m, 5H, H-21, N(1)-C*H_3_*, H_x_-3), 3.48–3.54 (m, 2H, H_y_-5, H_y_-3), 3.82 (s, 3H, C(16)-COOC*H_3_*), 4.18 (s, 1H, H-2), 5.16 (s, 1H, H-17), 5.35 (br d, *J* = 10.2 Hz, 1H, H-15), 5.51 (s, 1H, H-12), 5.96 (ddd, *J* = 10.2, 5,1, 1.3 Hz, 1H, H-14), 6.38 (s, 1H, H-9).

^13^C NMR (125.7 MHz, CDCl_3_) *δ* (ppm) 7.5 (C-18), 20.8 (C(17)-OC(O)*C*H_3_), 31.1 (C-19), 35.3 (N(1)-*C*H_3_), 42.2 (C-6), 42.8 (C-20), 50.3 (C-3), 50.7 (C-5), 51.5 (C-7), 52.9 (C(16)-COO*C*H_3_), 66.8 (C-21), 74.7 (C-17), 79.2 (C-16), 82.1 (C-2), 94.7 (C-12), 123.8 (C-9), 124.7 (C-14), 129.4 (C-15), 157.6 (C-8), 157.9 (C-13), 170.1 (C(17)-O*C*(O)CH_3_), 170.6 (C(16)-*C*OOCH_3_), 174.6 (C-11), 180.7 (C-10).

HRMS: M + H = 457.19636 (C_24_H_29_O_7_N_2_, Δ = –1.2 ppm). ESI-MS-MS (CID = 45%, rel. int. %): 439(23), 429(34), 415(34), 411(3), 397(100), 379(10), 369(9), 368(10), 347(6), 337(27), 295(15), 290(7), 190(7).

### 3.10. Epoxidation of Catharanthine *(**3**)*

*Method A. Without perchloric acid.* To a solution of catharanthine (**3**) (160 mg, 0.48 mmol) in dry methanol (10 mL), *m*-CPBA (184 mg, 0.82 mmol) was added, and the reaction mixture was stirred at room temperature for 1 h. The reaction mixture was diluted with 10% aqueous sodium carbonate (10 mL) and extracted with dichloromethane (3 × 10 mL). The combined organic phase was dried over magnesium sulfate and evaporated under reduced pressure. The residue was purified by preparative TLC (dichloromethane-methanol 4:1) and products **20** (57 mg, 34%) and **21** (62 mg, 35%) were isolated. *N*-oxide **20** is prone to rearrangement and transforms into isoxazolidine **22**, which is a stable compound.

**20** and **22**; mp 116–117 °C. TLC (dichloromethane-methanol 4:1); *R_f_* (*N*-oxide form (**20**)) = 0.76 and *R_f_* (neutral form (**22**)) = 0.90.

IR (KBr) 3378, 3185, 2960, 1735, 1460, 1435, 1237, 744 cm^−1^.

NMR: (chemical shifts might vary slightly with concentration, pH and the exact ratio of **20** and **22**)

*N*-oxide **20**:

^1^H NMR (499.9 MHz, CDCl_3_) *δ* (ppm) 1.04 (t, *J* = 7.4 Hz, 3H, H_3_-18), 1.58–1.62 (m, 1H, H_x_-17), 2.12–2.19 (m, 1H, H_x_-19), 2.44–2.50 (m, 1H, H_y_-19), 2.83–2.88 (m, 1H, H_y_-17), 2.92–2.96 (m, 1H, H-14), 2.96–3.02 (m, 1H, H_x_-6), 3.40–3.48 (m, 2H, H_x_-3, H_y_-6), 3.67 (s, 3H, C(16)-COOC*H_3_*), 3.74–3.79 (m, 1H, H_y_-3), 3.92 (ddd, *J* = 13.2, 8.3, 1.6 Hz, 1H, H_x_-5), 4.27–4.34 (m, 1H, H_y_-5), 4.72–4.74 (m, 1H, H-21), 6.07–6.10 (m, 1H, H-15), 7.06–7.09 (m, 1H, H-10), 7.11–7.15 (m, 1H, H-11), 7.21–7.24 (m, 1H, H-12), 7.41–7.43 (m, 1H, H-9), 7.88 (br s, 1H, NH-1).

^13^C NMR (125.7 MHz, CDCl_3_) *δ* (ppm) 10.4 (C-18), 19.8 (C-6), 28.3 (C-19), 30.0 (C-14), 32.3 (C-17), 51.0 (C-16), 53.2 (C(16)-COO*C*H_3_), 73.5 (C-3), 74.4 (C-21), 76.9 (C-5), 111.1 (C-12), 111.7 (C-7), 118.2 (C-9), 120.3 (C-10), 122.8 (C-11), 124.3 (C-15), 127.4 (C-8), 133.7 (C-2), 134.9 (C-13), 145.9 (C-20), 171.5 (C(16)-*C*OOCH_3_).

Isoxazolidine **22**:

^1^H NMR (499.9 MHz, CDCl_3_) *δ* (ppm) 1.14 (t, *J* = 7.5 Hz, 3H, H_3_-18), 1.98 (dd, *J* = 14.2, 6.1 Hz, 1H, H_x_-17), 2.18–2.26 (m, 1H, H_x_-19), 2.26–2.30 (m, 1H, H_y_-17), 2.36–2.45 (m, 1H, H_y_-19), 2.58 (dd, *J* = 10.6, 5.2 Hz, 1H, H_x_-3), 2.75–2.80 (m, 1H, H_x_-6), 2.94–3.01 (m, 1H, H_x_-5), 3.13–3.20 (m, 1H, H-14), 3.29–3.37 (m, 3H, H_y_-3, H_y_-5, H_y_-6), 3.77 (s, 3H, C(16)-COOC*H_3_*), 4.53 (d, *J* = 10.0 Hz, 1H, H-15), 6.19–6.20 (m, 1H, H-21), 7.00–7.04 (m, 1H, H-10), 7.10–7.13 (m, 1H, H-11), 7.24–7.26 (m, 1H, H-12), 7.39–7.42 (m, 1H, H-9), 8.67 (br s, 1H, NH-1).

^13^C NMR (125.7 MHz, CDCl_3_) *δ* (ppm) 11.3 (C-18), 23.7 (C-6), 27.4 (C-19), 32.5 (C-17), 39.9 (C-14), 46.7 (C-16), 53.1 (C(16)-COO*C*H_3_), 55.5 (C-3), 55.8 (C-5), 76.6 (C-15), 110.8 (C-12), 113.6 (C-7), 118.4 (C-9), 119.5 (C-10), 121.9 (C-21), 122.7 (C-11), 127.9 (C-2), 128.0 (C-8), 135.2 (C-13), 141.5 (C-20), 174.9 (C(16)-*C*OOCH_3_).

HRMS:

*N*-oxide **20**:

M+H = 353.18559 (C_21_H_25_O_3_N_2_, Δ = –1.1 ppm). ESI-MS-MS (CID = 45%, rel. int. %): 336(100), 321(7), 303(6), 294(4), 293(3), 275(2), 266(6), 229(2), 189(2), 171(2), 144(12).

Isoxazolidine **22**:

M+H = 353.18567 (C_21_H_25_O_3_N_2_, Δ = –0.9 ppm). ESI-MS-MS (CID = 45%, rel. int. %): 335(100), 321(36), 303(36), 294(22), 293(15), 275(11), 266(38), 171(5), 144(4).

**21**; mp 146–147 °C. TLC (dichloromethane-methanol 4:1); *R_f_* = 0.52.

IR (KBr) 3581, 3365, 2959, 1744, 1570, 1242, 774 cm^−1^.

^1^H NMR (499.9 MHz, DMSO-*d*_6_:CDCl_3_ = 1:1 + 5 *v/v*% CF_3_COOH) *δ* (ppm) 1.07 (t, *J* = 7.4 Hz, 3H, H_3_-18), 1.99–2.11 (m, 2H, H_x_-17, H_x_-19), 2.16–2.25 (m, 1H, H_x_-6), 2.25–2.34 (m, 1H, H_y_-19), 2.49–2.56 (m, 1H, H_y_-6), 2.87–2.93 (m, 1H, H_y_-17), 3.11–3.15 (m, 1H, H-14), 3.34–3.40 (m, 1H, H_x_-3), 3.67 (s, 3H, C(16)-COOC*H_3_*), 3.90–3.95 (m, 1H, H_y_-3), 4.14–4.29 (m, 1H, H_x_-5), 4.54–4.63 (m, 1H, H_y_-5), 5.59 (d, *J* = 1.4 Hz, 1H, H-21), 6.24–6.27 (m, 1H, H-15), 7.28–7.32 (m, 1H, H-10), 7.36–7.40 (m, 1H, H-11), 7.44–7.46 (m, 1H, H-12), 7.47–7.49 (m, 1H, H-9).

^13^C NMR (125.7 MHz, DMSO-*d*_6_:CDCl_3_ = 1:1 + 5 *v/v*% CF_3_COOH) *δ* (ppm) 10.2 (C-18), 26.9 (C-19), 29.0 (C-14), 30.0 (C-6), 34.6 (C-17), 52.3 (C-16), 53.3 (C(16)-COO*C*H_3_), 64.3 (br, C-3), 65.2 (br, C-5), 70.8 (C-21), 82.8 (C-7), 120.6 (C-12), 122.5 (C-9), 125.9 (C-15), 126.9 (C-10), 129.7 (C-11), 140.2 (br, C-8), 141.4 (C-20), 152.0 (C-13), 168.1 (C(16)-*C*OOCH_3_), 184.0 (C-2).

HRMS: M + H = 369.18063 (C_21_H_25_O_4_N_2_, Δ = –0.7 ppm). ESI-MS-MS (CID = 45%, rel. int. %): 351(16), 335(9), 324(29), 307(100), 292(5), 264(14), 205(3), 187(4), 160(6).

*Method B. Using perchloric acid.* To a solution of catharanthine (**3**) (247 mg, 0.73 mmol) in methanol (10 mL) and 72% perchloric acid (0.10 mL), *m*-CPBA (184 mg, 0.82 mmol) was added at 0 °C, and the reaction mixture was stirred at room temperature for 18 h. The reaction mixture was diluted with 10% aqueous sodium carbonate (10 mL) and extracted with dichloromethane (3x10 mL). The combined organic phase was dried over magnesium sulfate and evaporated under reduced pressure. The residue was purified by preparative TLC (dichloromethane-methanol 10:1) and products **23** (149 mg, 58%) and **24** (30 mg, 12%) were isolated.

Compound **23**; mp 87–89 °C. TLC (dichloromethane-methanol 10:1); *R_f_* = 0.55.

IR (KBr) 3436, 2960, 1741, 1459, 1222, 1078, 757 cm^−1^.

^1^H NMR (499.9 MHz, DMSO-*d*_6_) *δ* (ppm) 0.96 (t, *J* = 7.4 Hz, 3H, H_3_-18), 1.73 (dd, *J* = 13.2, 2.3 Hz, 1H, H_x_-17), 1.75–1.83 (m, 1H, H_x_-6), 1.87–1.96 (m, 2H, H_y_-6, H_x_-19), 2.07 (dqd, *J* = 16.1, 7.4, 2.0 Hz, H_y_-19), 2.52 (dt, *J* = 8.3, 2.6 Hz, 1H, H_x_-3), 2.55–2.58 (br m, 1H, H-14), 2.59 (br d, *J* = 8.3 Hz, 1H, H_y_-3), 2.73–2.78 (m, 1H, H_y_-17), 2.79–2.84 (m, 1H, H_x_-5), 3.50 (s, 3H, C(16)-COOC*H_3_*), 3.61 (ddd, *J* = 14.3, 12.4, 2.3 Hz, 1H, H_y_-5), 4.57 (~d, *J* = 1.1 Hz, 1H, H-21), 5.75–5.77 (m, 1H, H-15), 6.01 (d, *J* = 0.9 Hz, 1H, C(7)-O*H*), 7.18–7.22 (m, 1H, H-10), 7.28–7.32 (m, 1H, H-11), 7.33–7.36 (m, 1H, H-12), 7.36–7,39 (m, 1H, H-9).

^13^C NMR (125.7 MHz, DMSO-*d*_6_) *δ* (ppm) 11.2 (C-18), 25.9 (C-19), 30.7 (C-14), 32.9 (C-6), 39.2 (C-17), 46.7 (C-5), 47.6 (C-3), 52.0 (C(16)-COO*C*H_3_), 57.4 (C-16), 57.8 (C-21), 87.1 (C-7), 119.9 (C-12), 121.9 (C-9), 122.4 (C-15), 126.1 (C-10), 128.8 (C-11), 143.0 (C-8), 148.1 (C-20), 152.4 (C-13), 171.6 (C(16)-*C*OOCH_3_), 190.3 (C-2).

HRMS: M + H = 353.18553 (C_21_H_25_O_3_N_2_, Δ = –1.2 ppm). ESI-MS-MS (CID = 35%, rel. int. %): 335(80), 321(100), 303(3), 189(4), 171(8).

Compound **24**; mp 99–101 °C (decomp.). TLC (dichloromethane-methanol 10:1); *R_f_* = 0.13.

IR (KBr) 1730, 1693, 1618, 755 cm^−1^.

^1^H NMR (499.9 MHz, DMSO-*d*_6_:CDCl_3_ = 2:1 *(v/v*)) *δ* (ppm) 1.03 (t, *J* = 7.4 Hz, 3H, H-18), 1.71 (br d, *J* = 14.8 Hz, 1H, H_x_-6), 1.95 (br d, *J* = 13.3 Hz, 1H, H_x_-17), 2.06 (dqd, *J* = 16.4, 7.4, 1.6 Hz, H_x_-19), 2.30–2.43 (m, 3H, H_y_-19, H_y_-6, H_y_-17), 2.83 (br d, 1H, *J* = 10.8 Hz, H_x_-3), 2.90–2.94 (m, 1H, H-14), 3.03–3.08 (m, 2H, H_y_-3, H_x_-5), 3.24 (s, 3H, C(16)-COOC*H_3_*), 4.10 (td, *J* = 12.9, 3.2 Hz, 1H, H_y_-5), 4.92 (br s, 1H, H-21), 6.17 (br d, *J* = 6.4 Hz, 1H, H-15), 6.55–6.64 (br, 1H, NH-1), 6.72–6.77 (m, 1H, H-10), 6.79–6.82 (m, 1H, H-12), 7.37–7.42 (m, 1H, H-11), 7.49–7.52 (m, 1H, H-9).

^13^C NMR (125.7 MHz, DMSO-*d*_6_:CDCl_3_ = 2:1 (*v/v*)) *δ* (ppm) 10.7 (C-18), 23.4 (C-6), 26.5 (C-19), 28.9 (C-14), 30.3 (C-17), 44.7 (C-5), 50.3 (C-3), 51.9 (C-16), 52.0 (C(16)-COO*C*H_3_), 54.0 (C-21), 65.0 (C-2), 112.1 (C-12), 118.5 (C-10), 119.4 (C-8), 124.2 (C-9), 128.0 (C-15), 137.3 (C-11), 144.2 (C-20), 159.9 (C-13), 171.2 (C(16)-*C*OOCH_3_), 202.2 (C-7).

HRMS: M + H = 353.18512 (C_21_H_25_O_3_N_2_, Δ = –2.4 ppm). ESI-MS-MS (CID = 45%, rel. int. %): 335(3), 321(2), 276(2), 189(100), 172(6), 160(38), 146(23).

### 3.11. Reduction of the 7-Hydroxy Derivative of Catharanthine *(**23**)*

To a solution of 7-hydroxyindolenine catharanthine (**23**) (300 mg, 0.85 mmol) in methanol (20 mL), 700 mg of 10% Pd/C and NaBH_4_ (483 mg, 12.77 mmol) was added at 10 °C. The reaction mixture was stirred under an argon atmosphere for 30 min. After filtering the catalyst, a few drops of acetic acid was added, the filtrate was diluted with dichloromethane (50 mL) and washed with 10% aqueous sodium carbonate. The aqueous phase was extracted with dichloromethane (2 × 20 mL), the combined organic phase was washed with water (50 mL), dried over magnesium sulfate and evaporated under reduced pressure. The residue was purified by preparative TLC (dichloromethane-methanol 9:1) and catharanthine (**3**) was obtained (111 mg, 39%).

### 3.12. Coupling of the Catharanthine Derivative *(**24**)* with Vindoline *(**4**)*

Compound **24** (210 mg, 0.60 mmol) and vindoline (271 mg, 0.59 mmol) (**4**) were added to a mixture that consisted of water (21.3 mL), 1 *M* hydrochloric acid (1.1 mL) and 2,2,2-trifluoroethanol (2.2 mL). Under an argon atmosphere, FeCl_3_·6 H_2_O (802 mg, 2.97 mmol) was added. The reaction mixture was stirred at room temperature for 2 h. At 0 °C, sodium borohydride (24 mg, 0.63 mmol) in water (1.9 mL) was added dropwise. After 30 min of stirring, the pH was adjusted to 8 with cc. ammonium hydroxide. The reaction mixture was extracted with dichloromethane (2 × 60 mL), the combined organic phase was washed with water (100 mL), dried over magnesium sulfate and evaporated under reduced pressure. The residue was purified by preparative TLC (dichloromethane-methanol 6:1). A cationic vindoline trimer (**27**) (18 mg, 7%), a vindoline trimer ketone (**28**) (26 mg, 10%), and the *N*-methyl-spiro derivative of catharanthine (**29**) (24 mg, 11%) were obtained.

Compound **27**:

TLC (dichloromethane-methanol 7:1), *R_f_* = 0.27.

NMR: two signal sets in a ratio of ca. 3:2 (conformational isomers). Chemical shifts vary slightly with pH, temperature, and the composition of the NMR solvent. The three vindoline subunits of **27** are denoted as A, B, and B’. The two subunits that have the same constitution (vindoline-10-yl) are called B and B’.

Major signal set:

^1^H NMR (799.7 MHz, CD_3_OD:CD_3_CN:D_2_O = 1:1:1 (*v/v*)) *δ* (ppm) 0.35 (t, *J* = 7.4 Hz, 3H, H_3_-18B), 0.52 (t, *J* = 7.4 Hz, 3H, H_3_-18B’), 0.57 (t, *J* = 7.4 Hz, 3H, H_3_-18A), 1.15–1.20 (m, 1H, H_x_-19B’), 1.25–1.30 (m, 1H, H_x_-19B), 1.47–1.53 (m, 1H, H_x_-19A), 1.53–1.58 (m, 1H, H_y_-19A), 1.55–1.61 (m, 1H, H_y_-19B’), 1.58–1.63 (m, 1H, H_y_-19B), 1.62–1.67 (m, 1H, H_x_-6A), 2.02 (s, 6H, C(17B)-OC(O)C*H_3_*, C(17B’)-OC(O)C*H_3_*), 2.06 (s, 3H, C(17A)-OC(O)C*H_3_*), 2.15–2.20 (m, 1H, H_x_-6B’), 2.20–2.24 (m, 2H, H_y_-6A, H_y_-6B’), 2.26–2.30 (m, 2H, H_x_-6B, H-21B), 2.32–2.35 (m, 1H, H_x_-5B), 2.37–2.41 (m, 1H, H_y_-6B), 2.54–2.58 (m, 1H, H_x_-5B’), 2.64 (s, 3H, N(1B)-C*H_3_*), 2.65 (s, 1H, H-21B’), 2.67–2.71 (m, 1H, H_x_-5A), 2.71 (s, 3H, N(1B’)-C*H_3_*), 2.73–2.77 (m, 1H, H_x_-3B), 2.85–2.89 (m, 1H, H_x_-3B’), 2.94–2.98 (m, 1H, H_x_-3A), 3.12 (s, 1H, H-21A), 3.28 (s, 3H, N(1A)-C*H_3_*), 3.30–3.34 (m, 1H, H_y_-5A), 3.37–3.43 (m, 3H, H_y_-5B’, H_y_-5B, H_y_-3A), 3.45–3.53 (m, 2H, H_y_-3B’, H_y_-3B), 3.66 (s, 1H, H-2B), 3.69 (s, 1H, H-2B’), 3.73 (s, 3H, C(11B’)-OC*H_3_*), 3.74 (s, 3H, C(11B)-OC*H_3_*), 3.78 (s, 3H, C(16B’)-COOC*H_3_*), 3.79 (s, 3H, C(16B)-COOC*H_3_*), 3.83 (s, 3H, C(16A)-COOC*H_3_*), 3.97 (s, 3H, C(11A)-OC*H_3_*), 4.39 (s, 1H, H-2A), 5.08 (s, 1H, H-17A), 5.08–5.11 (m, 1H, H-15B), 5.19–5.22 (m, 1H, H-15B’), 5.32 (s, 1H, H-17B’), 5.38–5.41 (s, 1H, H-15A), 5.43 (s, 1H, H-17B), 5.79–5.82 (m, 1H, H-14B), 5.87–5.90 (m, 1H, H-14B’), 5.94–5.97 (m, 1H, H-14A), 6.27 (s, 1H, H-12B), 6.36 (s, 1H, H-12B’), 6.39 (s, 1H, H-12A), 6.42 (s, 1H, H-9B’), 6.63 (s, 1H, H-9B), 7.29 (s, 1H, H-9A).

^13^C NMR (201.1 MHz, CD_3_OD:CD_3_CN:D_2_O = 1:1:1 (*v/v*)) *δ* (ppm) 7.6 (C-18A), 9.2 (C-18B), 9.5 (C-18B’), 21.0 (C(17B)-OC(O)*C*H_3_, C(17A)-OC(O)*C*H_3_, C(17B’)-OC(O)*C*H_3_), 30.8 (C-19A), 31.9 (C-19B’), 32.7 (C-19B), 37.3 (N(1A)-*C*H_3_), 38.3 (N(1B’)-*C*H_3_), 38.7 (N(1B)-*C*H_3_), 43.2 (C-6A), 43.3 (C-20A), 44.0 (C-6B, C-6B’), 44.1 (C-20B’), 44.4 (C-20B), 49.2 (C-5A), 50.6 (C-3A), 51.6 (C-3B’), 51.9 (C-3B, C-7A), 52.1 (C-5B’), 53.3 (C(16B’)-COO*C*H_3_), 53.4 (C(16B)-COO*C*H_3_), 53.7 (C-7B), 53.8 (C-5B), 54.0 (C(16A)-COO*C*H_3_), 54.1 (C-7B’), 56.5 (C(11B’)-O*C*H_3_), 57.2 (C(11B)-O*C*H_3_), 59.3 (C(11A)-O*C*H_3_), 59.4 (C-10A), 63.4 (C-21A), 67.8 (C-21B’), 69.2 (C-21B), 76.8 (C-17A), 76.9 (C-17B’), 77.0 (C-17B), 80.1 (C-16B, C-16A), 80.5 (C-16B’), 83.8 (C-2B’), 84.0 (C-2B), 84.6 (C-2A), 91.3 (C-12A), 95.1 (C-12B’), 95.5 (C-12B), 115.9 (C-10B), 116.9 (C-10B’), 124.0 (C-9B’), 126.0 (C-14B), 126.1 (C-9B), 126.2 (C-14B’), 126.4 (C-14A), 126.5 (C-8B’), 126.6 (C-8B), 130.2 (C-15A), 130.6 (C-15B, C-15B’), 132.3 (C-8A), 145.9 (C-9A), 155.3 (C-13B), 155.7 (C-13B’), 159.9 (C-11B), 160.9 (C-11B’), 169.9 (C-13A), 172.0 (C(16A)-*C*OOCH_3_), 172.2 (C(17A)-O*C*(O)CH_3_), 172.8 (C(17B’)-O*C*(O)CH_3_), 173.0 (C(17B)-O*C*(O)CH_3_), 173.4 (C(16B’)-*C*OOCH_3_), 173.7 (C(16B)-*C*OOCH_3_), 190.6 (C-11A).

Minor signal set:

^1^H NMR (799.7 MHz, CD_3_OD:CD_3_CN:D_2_O = 1:1:1 (*v/v*)) *δ* (ppm) 0.29 (t, *J* = 7.4 Hz, 3H, H_3_-18A), 0.46 (t, *J* = 7.4 Hz, 3H, H_3_-18B’), 0.61 (t, *J* = 7.4 Hz, 3H, H_3_-18B), 0.85–0.89 (m, 1H, H_x_-19A), 1.09–1.14 (m, 2H, H_x_-19B, H_x_-19B’), 1.41–1.45 (m, 1H, H_y_-19A), 1.50–1.54 (m, 1H, H_y_-19B’), 1.57–1.61 (m, 1H, H_y_-19B), 2.00 (s, 3H, C(17A)-OC(O)C*H_3_*), 2.01 (s, 3H, C(17B)-OC(O)C*H_3_*), 2.02 (s, 3H, C(17B’)-OC(O)C*H_3_*), 2.07–2.11 (m, 2H, H_x_-6B’, H_x_-6B), 2.19–2.26 (m, 3H, H_y_-6B, H_x_-6A, H_y_-6B’), 2.43–2.47 (m, 1H, H_x_-5B’), 2.47–2.51 (m, 1H, H_y_-6A), 2.57 (s, 1H, H-21B’), 2.63–2.66 (m, 1H, H_x_-5B), 2.68 (s, 3H, N(1B’)-C*H_3_*), 2.72 (s, 3H, N(1B)-C*H_3_*), 2.77–2.80 (m, 1H, H_x_-5A), 2.79–2.83 (m, 1H, H_x_-3B’), 2.87 (s, 1H, H-21B), 2.90–2.96 (m, 2H, H_x_-3A, H_x_-3B), 2.98 (s, 1H, H-21A), 3.23 (s, 3H, N(1A)-C*H_3_*), 3.37–3.42 (m, 2H, H_y_-5B’, H_y_-5B), 3.42–3.45 (m, 1H, H_y_-5A), 3.44–3.49 (m, 3H, H_y_-3A, H_y_-3B, H_y_-3B’), 3.62 (s, 3H, C(11B’)-OC*H_3_*), 3.65 (s, 1H, H-2B’), 3.67 (s, 1H, H-2B), 3.73 (s, 3H, C(11B)-OC*H_3_*), 3.75 (s, 3H, C(16B)-COOC*H_3_*), 3.76 (s, 3H, C(16B’)-COOC*H_3_*), 3.80 (s, 3H, C(16A)-COOC*H_3_*), 4.01 (s, 3H, C(11A)-OC*H_3_*), 4.50 (s, 1H, H-2A), 4.95 (s, 1H, H-17A), 5.19 (s, 1H, H-17B), 5.22–5.26 (m, 2H, H-15B’, H-15A), 5.24 (s, 1H, H-17B’), 5.35–5.38 (m, 1H, H-15B), 5.87–5.90 (m, 1H, H-14B’), 5.90–5.93 (m, 2H, H-14A, H-14B), 6.20 (s, 1H, H-12A), 6.23 (s, 2H, H-12B, H-12B’), 6.52 (s, 1H, H-9B), 6.55 (s, 1H, H-9B’), 7.39 (br s, 1H, H-9A).

^13^C NMR (201.1 MHz, CD_3_OD:CD_3_CN:D_2_O = 1:1:1 (*v/v*)) *δ* (ppm) 7.6 (C-18A), 8.1 (C-18B), 8.7 (C-18B’), 20.8 (C(17A)-OC(O)*C*H_3_), 21.0 (C(17B)-OC(O)*C*H_3_), 21.1 (C(17B’)-OC(O)*C*H_3_), 31.4 (C-19B), 31.6 (C-19B’), 32.1 (C-19A), 37.2 (N(1A)-*C*H_3_), 38.2 (N(1B)-*C*H_3_), 38.3 (N(1B’)-*C*H_3_), 42.7 (C-6A), 43.4 (C-20A), 43.8 (C-20B), 43.9 (C-20B’), 44.3 (C-6B’), 44.9 (C-6B), 50.1 (C-5A), 50.7 (C-5B), 50.9 (C-3A), 51.2 (C-3B), 51.4 (C-7A), 51.6 (C-3B’), 51.9 (C-5B’), 53.2 (C(16B)-COO*C*H_3_), 53.3 (C(16B’)-COO*C*H_3_), 53.9 (C(16A)-COO*C*H_3_), 54.1 (C-7B’), 54.4 (C-7B), 56.5 (C(11B)-O*C*H_3_), 56.7 (C(11B’)-O*C*H_3_), 58.0 (C-10A), 59.5 (C(11A)-O*C*H_3_), 65.2 (C-21A), 66.1 (C-21B), 67.1 (C-21B’), 76.6 (C-17A), 76.9 (C-17B), 77.2 (C-17B’), 79.6 (C-16A), 80.8 (C-16B’), 81.2 (C-16B), 83.3 (C-2B), 83.4 (C-2B’), 84.9 (C-2A), 89.4 (C-12A), 94.7 (C-12B), 95.6 (C-12B’), 115.9 (C-10B), 117.3 (C-10B’), 124.3 (C-9B), 125.2 (C-8B), 125.3 (C-9B’), 125.4 (C-8B’), 125.7 (C-14B), 125.9 (C-14B’), 126.1 (C-14A), 130.1 (C-15A), 130.8 (C-15B’), 131.0 (C-15B), 132.5 (C-8A), 147.2 (br, C-9A), 154.6 (C-13B), 154.9 (C-13B’), 159.8 (C-11B), 160.7 (C-11B’), 169.9 (C-13A), 171.7 (C(17A)-O*C*(O)CH_3_), 172.1 (C(16A)-*C*OOCH_3_), 172.5 (C(17B)-O*C*(O)CH_3_), 172.9 (C(17B’)-O*C*(O)CH_3_), 173.0 (C(16B)-*C*OOCH_3_), 173.3 (C(16B’)-*C*OOCH_3_), 191.8 (C-11A).

HRMS: M + H = 1365.65951 (C_75_H_93_O_18_N_6_, Δ = 3.97 ppm). ESI-MS-MS (CID = 35%, rel. int. %): 1347(17), 1305(73), 1245(9), 1203(10), 1096(100), 1036(17).

Compound **28**:

TLC (dichloromethane-methanol 10:1), *R_f_* = 0.35.

IR (KBr) 3433, 1749, 1619, 1289, 1207, 1151, 1025, 821 cm^−1^.

NMR: The three vindoline subunits of **28** are denoted as A, B, and B’. The two subunits that have the same constitution (vindoline-10-yl) are called B and B’. A minor signal set (ca. 10%) can also be detected due to conformational isomerism. The assignment of the major signal set is given below.

^1^H NMR (799.7 MHz, DMSO-*d_6_*:CD_3_CN:D_2_O = 3:1:1 (*v/v*)) *δ* (ppm) 0.30 (t, *J* = 7.3 Hz, 3H, H_3_-18A), 0.43 (t, *J* = 7.3 Hz, 3H, H_3_-18B), 0.50 (t, *J* = 7.3 Hz, 3H, H_3_-18B’), 0.82–0.86 (m, 1H, H_x_-19B’), 0.92–0.96 (m, 1H, H_x_-19A), 1.21 (dq, *J* = 15.2, 7.3 Hz, 1H, H_x_-19B), 1.39–1.43 (m, 1H, H_y_-19A), 1.43–1.50 (m, 2H, H_y_-19B’ H_y_-19B), 1.91 (s, 3H, C(17A)-OC(O)C*H_3_*), 1.94 (s, 3H, C(17B’)-OC(O)C*H_3_*), 1.93–1.97 (m, 1H, H_x_-6B), 1.96 (s, 3H, C(17B)-OC(O)C*H_3_*), 2.08–2.16 (m, 3H, H_y_-6B, H_x_-6B’, H_y_-6B’), 2.16–2.20 (m, 2H, H_x_-5B, H_x_-6A), 2.31 (s, 1H, H-21B), 2.34–2.38 (m, 1H, H_y_-6A), 2.55–2.59 (m, 2H, H_x_-5A, H_x_-5B’), 2.58 (s, 3H, N(1B)-C*H_3_*), 2.61–2.65 (m, 8H, N(1B’)-C*H_3_*, H_x_-3B, N(1A)-C*H_3_*, H-21A), 2.73 (br s, 1H, H-21B’), 2.79 (br d, *J* = 16.4 Hz, 1H, H_x_-3A), 2.85 (br d, *J* = 16.7 Hz, 1H, H_x_-3B’), 3.23–3.27 (m, 1H, H_y_-5B), 3.29–3.35 (m, 2H, H_y_-5A, H_y_-5B’), 3.37–3.42 (m, 3H, H_y_-3B’, H_y_-3B, H_y_-3A), 3.49 (s, 1H, H-2B), 3.53 (s, 3H, C(11B)-OC*H_3_*), 3.55 (s, 1H, H-2B’), 3.58 (s, 3H, C(11B’)-OC*H_3_*), 3.65 (s, 3H, C(16A)-COOC*H_3_*), 3.67 (s, 3H, C(16B’)-COOC*H_3_*), 3.68 (s, 3H, C(16B)-COOC*H_3_*), 3.91 (s, 1H, H-2A), 4.97 (s, 1H, H-17A), 5.09 (s, 1H, H-12A), 5.10–5.12 (m, 2H, H-15A, H-17B’), 5.16 (br d, *J* = 9.6 Hz, 1H, H-15B), 5.20–5.23 (m, 2H, H-17B, H-15B’), 5.81–5.83 (m, 2H, H-14B, H-14A), 5.83–5.86 (m, 1H, H-14B’), 6.12 (s, 1H, H-12B), 6.18 (s, 1H, H-9B), 6.20 (s, 1H, H-12B’), 6.80 (s, 1H, H-9B’), 7.17 (s, 1H, H-9A).

^13^C NMR (201.1 MHz, DMSO-*d_6_*:CD_3_CN:D_2_O = 3:1:1 (*v/v*)) *δ* (ppm) 7.2 (C-18B’), 7.7 (C-18A), 8.1 (C-18B), 20.6 (C(17A)-OC(O)*C*H_3_), 20.77 (C(17B’)-OC(O)*C*H_3_), 20.81 (C(17B)-OC(O)*C*H_3_), 30.7 (C-19B’), 30.9 (C-19B), 31.2 (C-19A), 34.4 (N(1A)-*C*H_3_), 38.2 (N(1B)-*C*H_3_), 38.8 (N(1B’)-*C*H_3_), 42.6 (C-20A), 42.7 (C-20B’), 42.8 (C-6A), 43.0 (C-20B), 43.2 (C-6B), 44.4 (C-6B’), 50.3 (C-7A), 50.4 (C-3A), 50.5 (C-5B’), 50.6 (C-3B’), 50.9 (C-5A), 51.0 (C-3B), 51.8 (C-5B), 52.1 (C(16B’)-COO*C*H_3_), 52.3 (C(16B)-COO*C*H_3_), 52.5 (C(16A)-COO*C*H_3_), 52.9 (C-7B), 53.1 (C-7B’), 55.3 (C(11B)-O*C*H_3_), 55.4 (C(11B’)-O*C*H_3_), 59.6 (C-10A), 65.6 (C-21B’), 67.1 (C-21B), 67.2 (C-21A), 75.7 (C-17A), 76.1 (C-17B’), 76.2 (C-17B), 78.7 (C-16A), 79.5 (C-16B), 79.7 (C-16B’), 81.7 (C-2A), 82.6 (C-2B), 83.4 (C-2B’), 92.1 (C-12A), 93.8 (C-12B), 94.3 (C-12B’), 118.5 (C-10B’), 123.0 (C-10B), 123.36 (C-8B), 123.42 (C-8B’), 124.56 (C-14A), 124.67 (C-14B’), 124.73 (C-9B’), 124.88(C-9B), 124.89 (C-14B), 129.9 (C-15A), 130.3 (C-15B), 130.6 (C-15B’), 133.4 (C-8A), 140,0 (C-9A), 152.4 (C-13B), 152.6 (C-13B’), 158.3 (C-11B), 159.5 (C-11B’), 162.2 (C-13A), 170.1 (C(17A)-O*C*(O)CH_3_), 170.5 (C(17B’)-O*C*(O)CH_3_), 171.1 (C(17B)-O*C*(O)CH_3_), 171.79 (C(16A)-*C*OOCH_3_), 171.83 (C(16B’)-*C*OOCH_3_), 172.1 (C(17B)-*C*OOCH_3_), 199.1 (C-11A).

HRMS: [(M + 2H)/2]^2+^ = 676.32271 (C_74_H_92_O_18_N_6_, Δ = –0.2 ppm). ESI-MS-MS (CID = 45%, rel. int. %): 646(100), 637(14), 616(17), 595(45), 565(16), 542(39), 512(16), 457(16).

Compound **29**:

TLC (dichloromethane-methanol 7:1), *R_f_* = 0.11.

^1^H NMR (799.7 MHz, DMSO-*d*_6_:CD_3_CN:D_2_O = 3:1:1 (*v/v*)) *δ* (ppm) 1.02 (t, *J* = 7.3 Hz, 3H, H_3_-18), 1.93–1.97 (m, 1H, H_x_-6), 2.04–2.09 (m, 1H, H_x_-19), 2.07–2.10 (m, 1H, H_x_-17), 2.18–2.24 (m, 1H, H_y_-19), 2.27–2.31 (m, 1H, H_y_-17), 2.55–2.60 (m, 1H, H_y_-6), 2.92–2.95 (m, 1H, H_x_-3), 3.04 (s, 3H, N(4)-C*H_3_*), 3.14–3.16 (m, 1H, H-14), 3.23 (s, 3H, C(16)-COOC*H_3_*), 3.58–3.61 (m, 1H, H_x_-5), 3.65–3.68 (m, 1H, H_y_-3), 4.10–4.14 (m, 1H, H_y_-5), 4.94 (d, *J* = 1.6 Hz, 1H, H-21), 6.41–6.44 (m, 1H, H-15), 6.80–6.84 (m, 1H, H-10), 6.87–6.90 (m, 1H, H-12), 7.48–7.50 (m, 1H, H-9), 7.50–7.53 (m, 1H, H-11).

^13^C NMR (201.1 MHz, DMSO-*d*_6_:CD_3_CN:D_2_O = 3:1:1 (*v/v*)) *δ* (ppm) 10.5 (C-18), 25.0 (C-6), 27.1 (C-19), 28.2 (C-17), 29.1 (C-14), 52.6 (C-16), 52.9 (C(16)-COO*C*H_3_), 55.6 (N(4)-*C*H_3_), 56.8 (C-5), 60.5 (C-3), 63.5 (C-2), 63.7 (C-21), 112.8 (C-12), 118.9 (C-8), 119.2 (C-10), 124.6 (C-9), 130.7 (C-15), 138.9 (C-11), 140.8 (C-20), 160.8 (C-13), 169.9 (C(16)-*C*OOCH_3_), 213.1 (C-7).

HRMS: M + H = 367.20122 (C_22_H_27_O_3_N_2_, Δ = –1.1 ppm). ESI-MS-MS (CID = 55%, rel. int. %): 203(100), 160(7).

## 4. Conclusions

Halogenated 14,15-cyclopropanovindoline derivatives were prepared by Simmons–Smith reactions with iodoform and bromoform in the presence of diethylzinc. Reactions of dichlorocarbene and catharanthine (**3**), vindoline (**4**), vinblastine (**1**) and vincristine (**2**) resulted in unexpected products. In the case of VLB (**1**), an interesting ring-opened oxirane derivative (**15**) was obtained with two different methods. Our attempt to produce epoxidized monomeric alkaloids also led to anomalous derivatives without an oxirane ring. It could have been easily noticed that the presence or absence of perchloric acid had had a crucial role in the outcome of these reactions. Eventually, we were surprised to discover two similar vindoline trimers (**27**,**28**) in the coupling reaction of vindoline (**4**) and a spiro derivative of catharanthine (**24**). Only the *O*-methylated trimeric vindoline derivative (**27**) was managed to be obtained when vindoline (**4**) was put in itself into the conditions of the formerly mentioned coupling reaction attempt without a catharanthine derivative (**24**).
